# HGR-ViT: Hand Gesture Recognition with Vision Transformer

**DOI:** 10.3390/s23125555

**Published:** 2023-06-14

**Authors:** Chun Keat Tan, Kian Ming Lim, Roy Kwang Yang Chang, Chin Poo Lee, Ali Alqahtani

**Affiliations:** 1 Faculty of Information Science and Technology, Multimedia University, Jalan Ayer Keroh Lama, Melaka 75450, Malaysia; 1181101099@student.mmu.edu.my (C.K.T.); kychang@mmu.edu.my (R.K.Y.C.); cplee@mmu.edu.my (C.P.L.); 2 Department of Computer Science, King Khalid University, Abha 61421, Saudi Arabia; amosfer@kku.edu.sa; 3 Center for Artificial Intelligence (CAI), King Khalid University, Abha 61421, Saudi Arabia

**Keywords:** hand gesture recognition, sign language recognition, vision transformer, ViT, attention

## Abstract

Hand gesture recognition (HGR) is a crucial area of research that enhances communication by overcoming language barriers and facilitating human-computer interaction. Although previous works in HGR have employed deep neural networks, they fail to encode the orientation and position of the hand in the image. To address this issue, this paper proposes HGR-ViT, a Vision Transformer (ViT) model with an attention mechanism for hand gesture recognition. Given a hand gesture image, it is first split into fixed size patches. Positional embedding is added to these embeddings to form learnable vectors that capture the positional information of the hand patches. The resulting sequence of vectors are then served as the input to a standard Transformer encoder to obtain the hand gesture representation. A multilayer perceptron head is added to the output of the encoder to classify the hand gesture to the correct class. The proposed HGR-ViT obtains an accuracy of 99.98%, 99.36% and 99.85% for the American Sign Language (ASL) dataset, ASL with Digits dataset, and National University of Singapore (NUS) hand gesture dataset, respectively.

## 1. Introduction

Hand gestures are a primary mode of communication for humans, with sign language being a natural form of hand gesture used by the hearing-impaired community to communicate. Unfortunately, most people in society are unable to interpret sign language, creating a language barrier for the mute and deaf community. In general, there are two categories of hand gesture recognition: static hand gesture recognition and dynamic hand gesture recognition. Static hand gesture recognition [1,2,3,4,5,6,7,8] involves interpreting hand gestures in a stationary position, resulting in higher accuracy due to less susceptibility to environmental factors. On the other hand, dynamic hand gesture recognition [9,10,11,12,13] recognises gestures with complex movements and temporal dynamics, which are more intuitive, natural, and versatile. However, static hand gestures have limitations in the variety of complex hand gestures that can be recognised. In view of this, this work is focusing on static hand gesture recognition. Static hand gesture recognition can be divided into two types: vision-based and wearable device-based. Vision-based static hand gesture recognition captures hand gestures using a camera, while wearable device-based static hand gesture recognition acquires hand gestures using wearable devices such as data gloves. The main focus of this work will be on vision-based static hand gesture recognition.

Over the years, researchers have proposed different methods to address the problem of static hand gesture recognition. These methods fall into two categories: hand-crafted approaches and deep learning approaches. Hand-crafted approaches, such as Scale Invariant Feature Transform (SIFT) [14], Histogram of Oriented Gradient (HOG) [15], and Discrete Wavelet Transform (DWT) [16], extract features from hand gesture images using manual feature extraction methods before passing them to a classifier. These methods are generally more time-consuming compared to deep learning approaches, and they require trial and error processes to find the best features for the specific task, which also consumes a lot of computational resources. Moreover, hand-crafted approaches may be biased towards the domain expert’s understanding, resulting in extracted features that may not accurately represent the most important or relevant features for the classification task, negatively impacting performance.

In contrast, deep learning approaches [17,18,19,20,21] extract features using hidden layers of neural networks, such as convolutional neural networks (CNN) and artificial neural networks (ANN). The extracted features can be classified by the same neural network or passed to another neural network for classification. Deep learning approaches extract more significant features, thereby improving classification performance. However, while deep learning approaches have achieved better performance than hand-crafted approaches, there is still room for improvement or optimization in hand gesture recognition. The proposed HGR-ViT addresses this issue by utilising the Vision Transformer model with a self-attention mechanism. With the self-attention mechanism, it can enhance the recognition performance by capturing complex relationships between image patches. This is particularly useful in addressing the challenges posed by visually similar hand gestures. Additionally, the proposed HGR-ViT utilises early stopping and adaptive learning rate to prevent overfitting and reduce generalisation error during the neural network training process.

The main contributions of this paper are as follows:

- The Vision Transformer model leverages the self-attention mechanism to capture complex relationships between the image patches which enables it to better handle the similarity problem between hand gestures as well as variations in pose, lighting, background and occlusions.
- Early stopping and adaptive learning rate are introduced to prevent overfitting and reduce generalisation error.
- Evaluating the proposed HGR-ViT method on three benchmark hand gesture datasets, including American Sign Language (ASL), ASL with Digits, and National University of Singapore (NUS) hand gesture datasets, and achieving promising performance on all three datasets.

## 2. Related Works

In general, static hand gesture recognition can be categorized into two approaches: hand-crafted and deep learning. Hand-crafted approaches aim to extract features from hand gesture images using manual feature extraction methods before being classified by classifiers. Scale Invariant Feature Transform (SIFT) was used as the feature extraction method in [14,22,23]. In [14], SIFT was used to extract the features, followed by a sparse autoencoder neural network for the classification of hand gesture images. A similar work [22] proposed to utilise SIFT as feature extraction and used a feedforward neural network to classify the hand gestures. The authors of [23] proposed a slightly different approach where Histogram of Oriented Gradient (HOG) and SIFT feature extractors were used together to extract the features which were then fused and classified by k-nearest neighbors (KNN). HOG was used to determine intensity distribution and local edges while the SIFT was utilised to capture the gradient orientations with weighted magnitude values. Later, HOG took over as the feature extraction method of choice by the researchers of [15,24] as it involves fewer computations and is more robust to geometric transformations such as scaling and rotation when compared to SIFT. The authors of [24] used Local Binary Pattern (LBP) and HOG together for feature extraction and Adaptive Boosting (AdaBoost) for classification of hand gestures. They found that the combination of both feature extractors was more robust against nonlinear illumination and image blurring and showed an improved recognition rate compared to using them individually. A later work [15] also proposed HOG for feature extraction of hand gesture images.

Later, Discrete Wavelet Transform (DWT) emerged as a replacement for HOG as DWT is able to capture both local and global features of an image. It is also more robust to noise and illumination changes than HOG which is crucial in hand gesture recognition. The work of [25] determined the DWT coefficients using F-ratio to reduce the dimensionality of the feature vector, resulting in a more efficient and faster recognition of hand gesture images. Similarly, Ref. [16] utilised DWT for feature extraction in hand gesture recognition. They created a characteristic matrix for each image by averaging the values in the contour pixel row and column pair, which produced four different sub-bands. The extracted features were then passed to a Hidden Markov Model (HMM) for classification. Recently, Ref. [26] proposed a two-dimensional DWT for feature extraction, Speed Up Robust Feature (SURF) for key point detection, and Bag of Feature (BoF) for feature space conversion. Then, *k*-means clustering was used to cluster the key points before passing them to a Support Vector Machine (SVM) for classification. Hand-crafted approaches are time-consuming and require trial and error to find the best feature extraction method for hand gesture recognition. Additionally, these methods can be biased towards the domain expert or researcher’s intuition, which may not always represent the most relevant or important features. Moreover, these methods have limited generalisability since they are designed for specific tasks and may not be able to generalize for other tasks.

In view of this, researchers have begun utilising deep learning approaches such as CNNs [1,2,3,4,6,17,27,28,29,30,31,32] and ANNs [33] over conventional hand-crafted methods. Deep learning approaches have the ability to automatically discover complex and important features through their hidden layers, saving time and reducing bias during feature extraction. Moreover, these models can generalize well to new data as they learn features that are useful for a variety of tasks, rather than relying on manually designed hand-crafted features. In [17], a parallel CNN was proposed for hand gesture recognition that combined the predictions of an RGB-CNN and Depth-CNN. The RGB-CNN processed RGB hand gesture images, while the Depth-CNN processed depth hand gesture images. The final prediction was determined by combining the predictions from both CNNs. In [27], the authors describe a study that explores the use of image processing techniques and convolutional neural networks (CNNs) to classify static hand gestures with invariance features. In addition, in [31], the authors proposed a six-layer CNN model that consists of conv1, conv2, SoftMax, max pooling, and fully connected layers to recognise hand gestures. Additionally, digital image processing techniques are utilised to detect a better region of interest that contains the hand sign and reduce errors caused by complex image backgrounds and variable illumination. Later, an adapted deep CNN [28] was proposed, with tuning of initialization and L2 regularization. The authors used uniform He initialization for ReLU layers and uniform Xavier initialization for the SoftMax layer of their proposed CNN model. L2 regularization was used to penalize large magnitude weights and reduce the model’s complexity. Moreover, in [29], a Directed Acyclic Graph (DAG)-CNN network architecture for recognising hand gestures is proposed. The Inception architecture is utilised to increase the network’s depth and learn more features for each gesture category, compensating for variations in lighting and noise.

The authors of [1] proposed a method for hand gesture recognition that employed transfer learning and fine-tuning of a pre-trained VGG19 CNN model. Two VGG19 models were trained, one for RGB images and one for depth images from an augmented ASL dataset. Then, the color and depth information from both models were concatenated for classification. Similarly, Ref. [2] adopted a pre-trained AlexNet CNN model with optimization using the Artificial Bee Colony (ABC) algorithm. In [30], a two-stage hand gesture recognition named HGR-Net was proposed, which involved training of three CNN models. In the first stage, a CNN model was trained for hand gesture segmentation. In the second stage, two CNN models were trained on the segmented image and the original image, respectively. The outputs of both models were fused using a fusion function and summed up to obtain the final prediction. A recent work by Mujahid et al. [4] proposed a model for hand gesture recognition using You Only Look Once (YOLO) v3 and DarkNet-53 CNN. Later, Tan et al. [3] proposed a CNN-SPP architecture that utilised Spatial Pyramid Pooling (SPP) to capture more spatial information, allowing the network to generalize to complex features. Additionally, Tan et al. introduced an enhanced version of DenseNet, called EDenseNet, in [6]. The modification was made to the transition layer by changing the first layer of the transition layer and adding a new layer before the pooling layer, resulting in better generalisation. Recently, the authors of [32] introduced a new method that utilises a standard RGB camera to extract 21 landmarks on the hand and focuses on the cloud of 3D reference points. They trained the network on hand KeyPoints and developed a new network based on the PointNet architecture, which has few hidden layers, allowing for direct CPU usage.

Most existing works in hand gesture recognition rely on CNNs due to their high performance, but they require a large number of training samples and fail to encode the precise position and orientation of objects. In light of these limitations, this paper proposes leveraging the ViT model, which has the ability to propagate information clearly from lower to higher levels. Additionally, the proposed HGR-ViT model can capture the position and orientation of complex hand gestures through positional embeddings. Furthermore, the HGR-ViT model obtains global information from the hand gesture images with the self-attention mechanism. Table 1 below provides a summary of related works in hand gesture recognition.

## 3. Hand Gesture Recognition with Vision Transformer (HGR-ViT)

**Figure 1:**
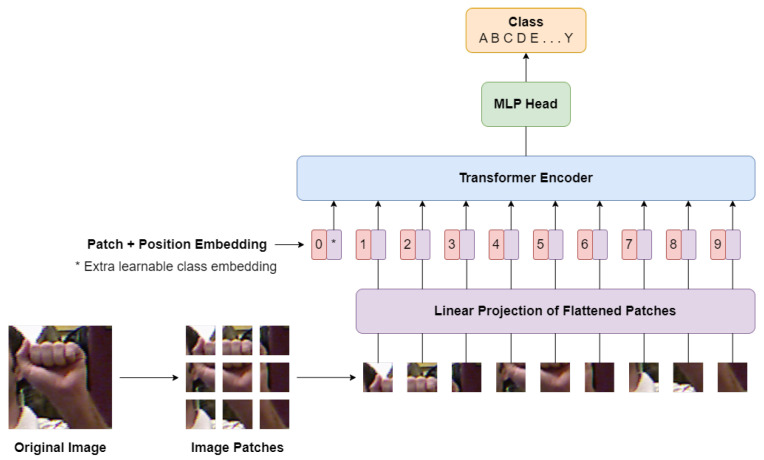
Architecture of the proposed HGR-ViT.

In this paper, we propose HGR-ViT, a Vision Transformer model for hand gesture recognition. The input images undergo a preprocessing step where they are resized to a fixed size and normalized. The resized images are then partitioned into non-overlapping patches and treated as a sequence of pixel values. To embed the patch sequences into a lower-dimensional space, we use a trainable linear projection layer. To retain the spatial information, each patch embedding is augmented with a learnable positional encoding. The patch embeddings and positional encodings are then processed by a stack of Transformer encoder layers, enabling the model to capture interactions between patches. Finally, the output of the last Transformer encoder layer is fed into a linear projection layer and softmax activation function to obtain the class probabilities. The entire model is trained end-to-end using supervised learning and cross-entropy loss. Figure 1 illustrates the architecture of the proposed HGR-ViT, including the patch embedding, positional encoding, Transformer encoder, and classification head. Additionally, Algorithm 1 outlines the training procedure for the proposed method.

**Algorithm 1** Algorithm of the training procedure of the proposed HGR-ViT**Require:**
Hand gesture training data *D_train_*
  1:  **for** *e* epochs **do**  2:        **for** *b* batch_size **do**  3:             *x,*
*y*←*D_train_*  4:             vit_model(*x*,*y*)  5:        **end for**  6:        Get es_monitor, es_patience, lr_monitor, lr_patience  7:        **if** es_monitor < self.best and es_patience ⩾ self.wait **then**  8:            **break**  9:        **end if**10:        **if** lr_monitor < self.best and lr_patience ⩾ self.wait and lr > min_delta **then**11:            Update learning rate12:        **end if**13:   **end for**14:   return vit_model

The Vision Transformer (ViT) is a neural network model adapted from the Transformer architecture, which is commonly used in Natural Language Processing (NLP) tasks such as text classification and translation. ViT was developed by the Google Research Brain Team and was first published in [34]. Unlike traditional convolutional neural networks (CNNs), ViT processes the image as a sequence of tokens, similar to how NLP processes text. This approach allows ViT to scale to larger image sizes and generalize better to different tasks without requiring task-specific modifications to the architecture. The authors found that ViT outperforms state-of-the-art CNN models while using only a quarter of the computational resources. However, ViT has a weaker inductive bias regarding translation equivariance and locality compared to CNN, which limits its ability to generalize well with smaller amounts of data. To overcome this limitation, ViT requires training on large datasets with at least 14 million images. If trained on smaller datasets, ViT’s performance is inferior to other CNN alternatives such as ResNet or EfficientNets. Therefore, in this research, we use the ViT model pre-trained on ImageNet as it is trained on a sufficiently large dataset to surpass the performance of state-of-the-art CNNs. The ViT model comes in various sizes and patch sizes. For this research, we adopted the ViT-Base 32 (ViT-B32) model, which has 86 million parameters and a patch size of 32×32.

To ensure that the hand gesture image can be divided into patches of size 32×32, it is first resized to a dimension of 256×256. This particular dimension is chosen because it has been found that fine-tuning the ViT at a higher resolution than the pre-training dataset (ImageNet-21k at 224×224 image dimension) can be more beneficial. Using higher resolution images while maintaining the same patch size results in a larger effective sequence length, which can improve performance. Once resized, the hand gesture image is partitioned into fixed-size patches. The image with height *H*, width *W*, and *C* channels is then reshaped into a sequence of flattened 2D patches, represented by $x_{P}\in R^{N\times (P^{2}\cdot C)}$. Here, $N=\frac{HW}{P^{2}}$ denotes the number of patches as well as the effective sequence length for the transformer, while (P,P) represents the resolution of each image patch.

### 3.1. Linear Projection of Flattened Patches

Before passing the patches into the Transformer Encoder blocks, they undergo a linear projection process. Each patch is first flattened into a vector $x_{p}^{n}$ of length $P^{2}\times C$, where n=1,…,N. Next, a trainable embedding matrix *E* maps the flattened patches to *D* dimensions to generate a sequence of embedded image patches. To introduce positional information and facilitate learning, the patch embeddings are augmented with one-dimensional positional embeddings $E_{pos}$, which are learned during the training process. To represent the classification output *y*, a learnable class embedding $x_{class}$, similar to the class token in Bidirectional Encoder Representations from Transformers (BERT), is prepended to the sequence of embedded image patches. The output of the linear projection process, $z_{0}$, can be represented using the following equation:

(1) $$z0=\left[x\mathrm{class};x_{p}^{1}E;x_{p}^{2}E;\ldots ;xp^{N}E\right]+Epos,$$

where $E\in R^{\left(P^{2}\cdot C\right)\times D}$ and $E_{pos}\in R^{(N+1)\times D}$.

**Figure 2:**
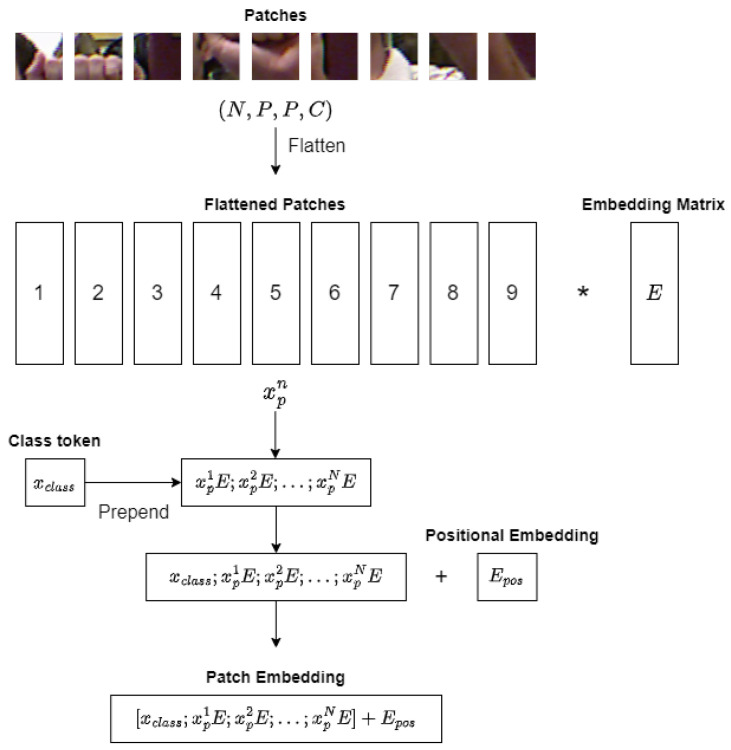
Linear projection process of ViT (* denotes multiplication).

The formation process of patch embeddings is illustrated in Figure 2. The patch embeddings serve as input to the transformer encoder, which allows ViT to effectively capture global patterns and dependencies in the image while also maintaining some spatial information through the use of patches.

### 3.2. Transformer Encoder

**Figure 3:**
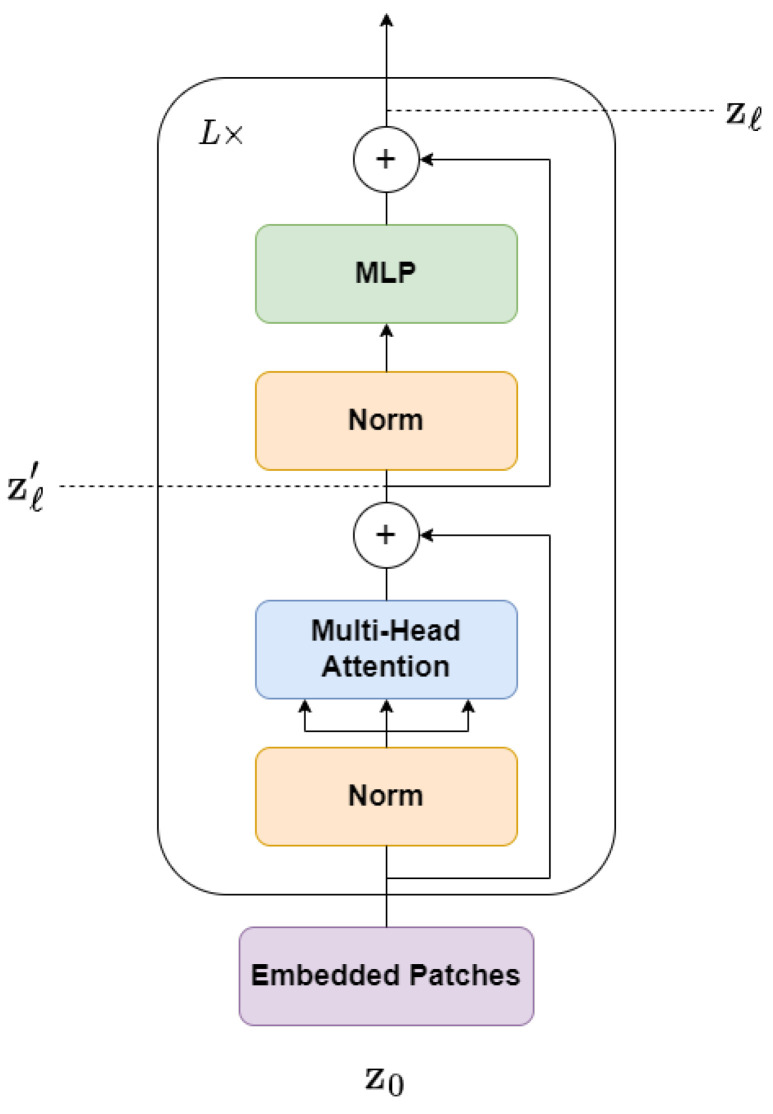
Architecture of the Transformer Encoder.

The Transformer encoder is an important component of the Transformer model. It is composed of a stack of *L* layers, each consisting of two sublayers: a Multi-head Self-Attention (MSA) layer and a position-wise feedforward layer, also known as a Multi-Layer Perceptron (MLP). These sublayers are arranged sequentially, where the output of each layer serves as the input to the next layer, as shown in Figure 3.

At each layer *ℓ*, the input sequence from the previous layer $z_{\ell -1}$ is normalized using Layer Normalization (LN), which independently normalizes the inputs across the features dimension for each example. This improves the stability of the model representations and generalization performance. The output of LN is passed through the MSA layer, and the resulting sequence is normalized again using LN. Finally, the output of the second LN is passed through the MLP layer, which produces a set of updated patch embeddings. Residual connections are added to the MLP layer to address the vanishing gradients problem, enabling the model to learn residual functions. The process flow in the Transformer Encoder block can be represented using the following equations:

(2) $$z\ell^{\prime }=\mathrm{MSA}\left(\mathrm{LN}\left(z\ell -1\right)\right)+z_{\ell -1},\;\ell =1\cdots L$$

(3) $$z\ell =\mathrm{MLP}\left(\mathrm{LN}\left(z\ell^{\prime }\right)\right)+z_{\ell }^{\prime },\;\ell =1\cdots L$$

In summary, the Transformer encoder uses self-attention mechanisms to capture global dependencies between input tokens, and MLPs to process the resulting representations. The residual connections and layer normalization ensure efficient training and better performance.

- Multi-head Self-Attention (MSA)

The MSA (Multi-Head Self-Attention) layer is a critical component of the Transformer Encoder block in the ViT (Vision Transformer) model. This layer enables the model to capture the contextual relationships between different parts of an image by analyzing the interactions between the patch embeddings. To achieve this, the MSA layer utilises the self-attention mechanism, which allows the model to assign varying importance to different elements in a sequence based on their relationships with other elements. In the case of ViT, the self-attention mechanism is employed to weigh the significance of different patch embeddings based on their relationships with other patches in the image. The MSA layer in ViT is referred to as “multi-head” because it consists of multiple self-attention heads, each of which learns to attend to different parts of the input.

**Figure 4:**
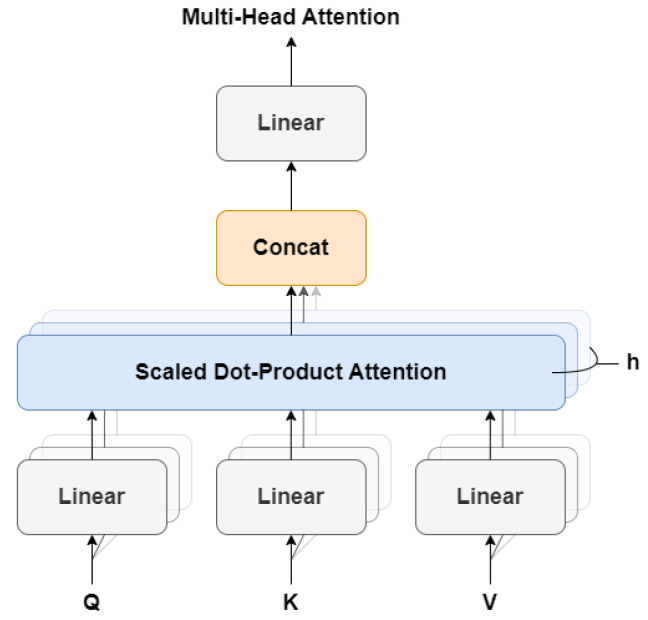
Architecture of the Multi-Head Self-Attention.

Each self-attention head in the MSA layer comprises three fully connected layers that are responsible for computing the queries (Q), keys (K), and values (V) for the attention mechanism. The queries, keys, and values are then utilised to compute the attention scores, which determine the importance of each patch embedding, based on its relationships with other patches. These attention scores are then used to weight the values, producing a weighted sum that represents the output of the self-attention mechanism. The MSA layer is composed of several components, including query, key, and value projections, dot product attention, multi-head attention, concatenation, and linear projection layers, as illustrated in Figure 4.

To begin with, the MSA layer takes an input sequence of patch embeddings $z=x_{1},x_{2},\cdots ,x_{n}$, where each patch embedding has a dimension of *d*. The MSA layer first applies linear projections to the input sequence to generate query, key, and value matrices *Q*, *K*, and *V*. These matrices have a dimension of d×h, where *h* represents the number of heads. This is achieved using learnable weight matrices $W_{q}$, $W_{k}$, and $W_{v}$, respectively, which form the equation:

(4) $$Q=zW_{q},\;K=zW_{k},\;V=zW_{v}$$

Next, the self-attention mechanism is applied to the queries, keys, and values matrices. The attention scores are computed using the dot product between the queries and keys, scaled by the square root of the dimension of the queries and keys:

(5) $$\mathrm{Attention}\left(Q,K,V\right)=\mathrm{softmax}\left(\frac{QK^{T}}{\sqrt{\frac{d}{h}}}\right)V$$

Here, the softmax function is applied along the last dimension of the attention scores, and the output is a weighted sum of the values, where the weights are determined by the attention scores. Finally, the outputs of the self-attention mechanism are concatenated across the heads dimension and linearly projected back to the original dimension *d*. This is achieved using a learnable weight matrix $W_{o}$, resulting in the final output of the MSA layer:

(6) $$\mathrm{MSA}\left(z\right)=\mathrm{concat}\left({\mathrm{Attention}}_{1},{\mathrm{Attention}}_{2},\cdots ,{\mathrm{Attention}}_{h}\right)W_{o}$$

- Multi-Layer Perceptron (MLP)

**Figure 5:**
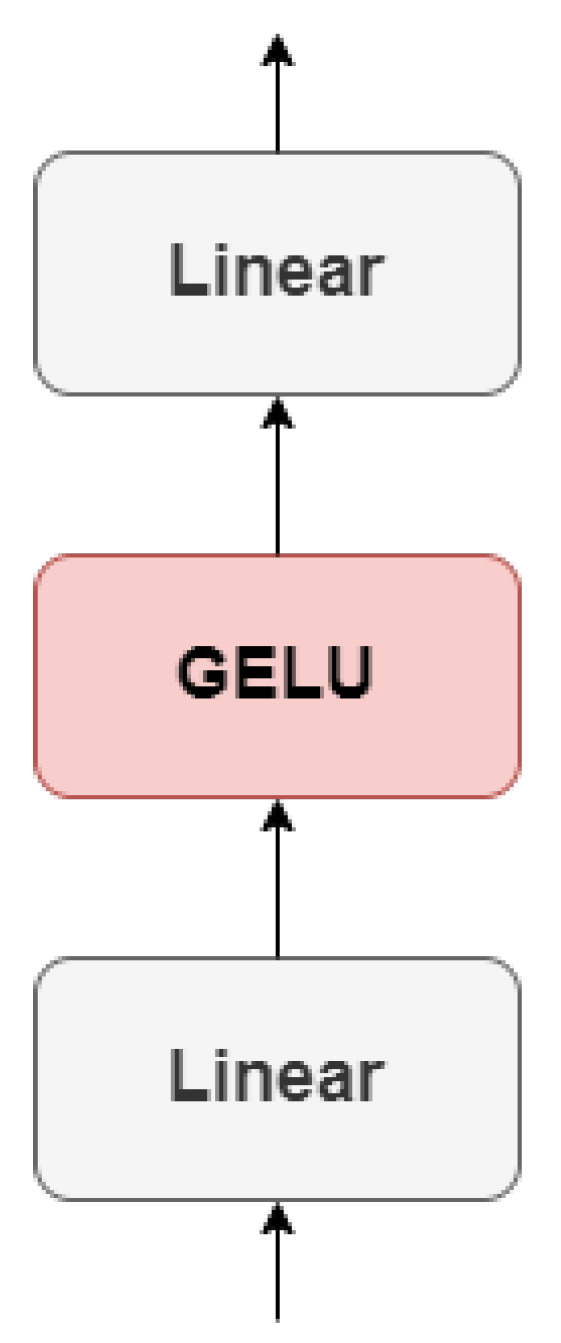
Architecture of the Multi-Layer Perceptron.

The ViT encoder includes an MLP block that follows the MSA layer. The MLP block is responsible for transforming the representations obtained from the self-attention layer into a higher-level feature space. It comprises two linear transformations separated by a non-linear activation function, as shown in Figure 5.

To begin, the patch embeddings *X* are multiplied with a learnable weight matrix $w_{1}$ in the first linear transformation. The output is then added with a bias $b_{1}$, producing ${\mathrm{MLP}}_{1}$ of shape (batch_size, num_patches, mlp_hidden_size):

(7) $${\mathrm{MLP}}_{1}=zw_{1}+b_{1}.$$

Afterwards, a non-linear activation function called Gaussian Error Linear Unit (GELU) is applied to the output, producing ${\mathrm{MLP}}_{2}$ of shape (batch_size, num_patches, mlp_hidden_size):

(8) $${\mathrm{MLP}}_{2}=\mathrm{GELU}\left({\mathrm{MLP}}_{1}\right).$$

The GELU function introduces non-linearity to the model, allowing it to learn complex and non-linear relationships between the input and output features, as shown in Equation (9):

(9) $$\mathrm{GELU}\left(x\right)=xP\left(X\leq x\right)=x\Phi \left(x\right)=x\cdot \frac{1}{2}\left[1+\mathrm{erf}\left(x/\sqrt{2}\right)\right],$$

where erf is the error function and P(X≤x) is the cumulative distribution function of a Gaussian distribution with mean 0 and variance 1 evaluated at *x*. ${\mathrm{MLP}}_{2}$ is then multiplied with another learnable weight matrix $w_{2}$ in the second linear transformation. The output is then added with a bias $b_{2}$, producing ${\mathrm{MLP}}_{3}$ of shape (batch_size, num_patches, mlp_hidden_size):

(10) $${\mathrm{MLP}}_{3}={\mathrm{MLP}}_{2}w_{2}+b_{2}.$$

After the MLP layer, another MSA layer and MLP layer follow in the ViT encoder block. This process is repeated *L* times to learn increasingly complex and abstract features of the input image.

At the end of the encoder block, the encoder selects the first token of the sequence $z_{L}^{0}$ and generates the image representation *y* by performing layer normalization, which is represented by:

(11) $$y=\mathrm{LN}\left(z_{L}^{0}\right).$$

The output *y* is then passed through a pooling layer to obtain a single feature vector representation of the entire image. The output is then passed through a flatten layer to convert the multi-dimensional input tensor into a one-dimensional vector. Finally, the outputs are passed through a batch normalization layer to improve the performance and stability of the network before passing through a fully-connected layer with a SoftMax function to perform the final classification.

During the training process, the Rectified Adam optimizer is used to efficiently update the model parameters. For multi-class classification problems, the categorical cross-entropy loss function is employed to measure the difference between the predicted probability distribution and the true probability distribution of the target variable. The SoftMax probabilities and labels are used to compute the loss function, as shown in the equation below:

(12) $$L_{CE}=-\sum_{i=1}T_{i}\log\left(S_{i}\right)$$

where *S* represents the SoftMax probabilities and *T* represents the labels. To prevent overfitting and improve model performance, early stopping and adaptive learning rate callback methods are also employed during the training process.

### 3.3. Early Stopping

Early stopping is an important technique used in deep learning to improve model generalisation by preventing overfitting. The technique involves monitoring the performance of the model on a selected metric during training and stopping the training process when the performance on the selected metric begins to degrade. In this research, we achieved this by comparing the current training set performance with the best training set performance recorded during training. If the current performance has not improved for a certain number of epochs, training is stopped, and the weights of the best performing model are returned.

The idea behind early stopping is that as the model trains, it becomes increasingly better at fitting the training data, but may begin to overfit and perform poorly on the training set. By monitoring the performance of the selected metric, we can stop the training process before the model overfits, thereby preventing it from learning spurious patterns in the training data that do not generalise well to new, unseen data.

In summary, early stopping is a powerful technique to improve the generalisation performance of deep learning models. By monitoring the performance of the selected metric and stopping the training process before overfitting occurs, we can prevent the model from learning spurious patterns in the training data that do not generalise well to new, unseen data. The technique can be customised with different hyperparameters to achieve optimal results.

### 3.4. Adaptive Learning Rate

Adaptive learning rate or reduce learning rate on plateau is a technique used to modify the learning rate of a neural network during the training process. The purpose of adaptive learning rate is to help in the convergence of the model so that the model does not converge too fast that it overshoots the local minimum and not too slow that training takes a long time which also helps in overcoming the overfitting problem. Adaptive learning rate adjusts the learning rate to optimize the training process.

Adaptive learning rate reduces the learning rate when a certain metric, such as the validation loss or accuracy, has stopped improving for a set number of epochs. This technique is useful when the learning rate is too high, causing the model to overshoot the local minimum and become stuck, or too low, causing slow convergence and an increased risk of overfitting.

The adaptive learning rate can be customized using its hyperparameters. The monitor parameter determines the metric to be monitored, such as validation loss or accuracy. The factor parameter determines the factor by which the learning rate is reduced when the monitored quantity has stopped improving. The patience parameter determines the number of epochs with no improvement in the monitored quantity before the learning rate is reduced. The mode parameter determines whether the monitored quantity should be increasing or decreasing to trigger the learning rate reduction. Finally, the minimum learning rate parameter sets a lower bound for the learning rate, preventing it from becoming too small.

Mathematically, the adaptive learning rate can be represented as follows:

(13) $$\begin{array}{cc} \; & \mathrm{if}\;M_{t-p}\leq M_{t}:\;\\ \; & \;LR_{t+1}=LR_{t}\times f\;\\ \; & \mathrm{else}:\;\\ \; & \;LR_{t+1}=LR_{t} \end{array}$$

where $LR_{t}$ be the learning rate at epoch *t*, and $M_{t}$ be the monitored quantity at epoch *t*. If $M_{t}$ does not improve for *p* epochs, the learning rate is reduced by a factor of *f*. *p* is the patience parameter, which determines the number of epochs with no improvement in the monitored quantity before the learning rate is reduced.

## 4. Experiment and Analysis

In this paper, three benchmark datasets are used to evaluate the proposed HGR-ViT: the American Sign Language (ASL) dataset, ASL with digits dataset, and NUS hand gesture dataset. Additionally, the performance of the proposed HGR-ViT is compared with several existing works.

### 4.1. Datasets

**Figure 6:**
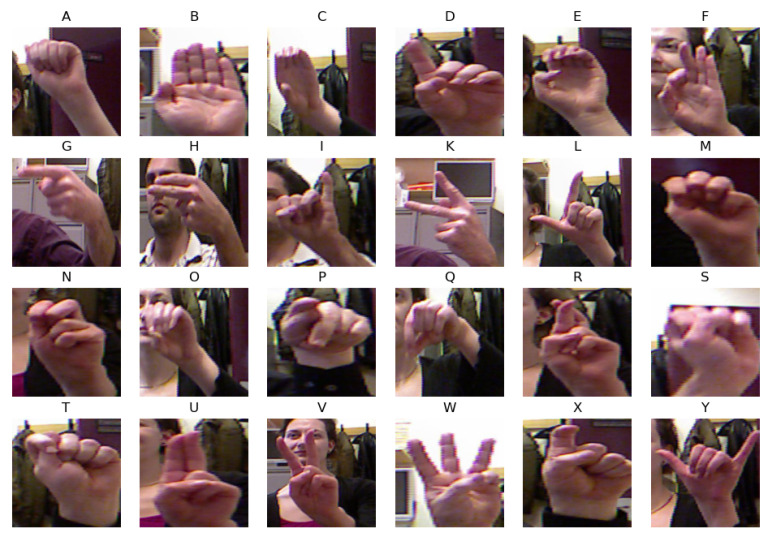
Image samples for each class from the ASL dataset.

The ASL dataset is composed of 65,774 images of 24 static hand gestures representing alphabets from A to Y. It was created by the authors of [35] and includes variations signed by five different signers. However, the dataset does not contain dynamic hand gestures such as J and Z. Figure 6 displays the images of each class, where the alphabet W has the highest number of samples at 6221 and the alphabet F has the lowest at 5235.

**Figure 7:**
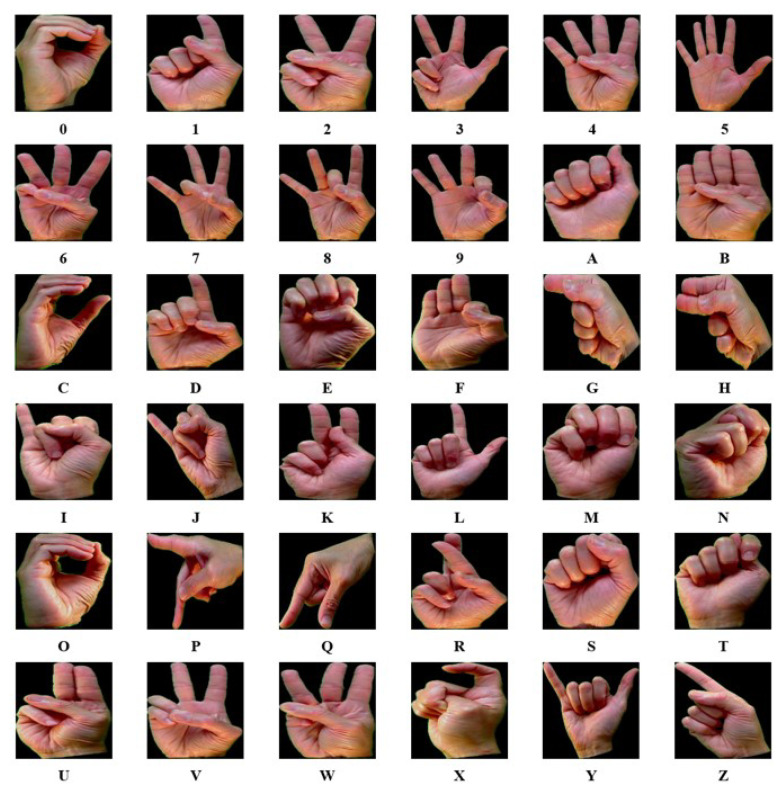
Image samples for each class from the ASL with digits dataset.

The ASL with digits dataset, introduced in [36], consists of 36 hand gesture classes, including alphabets from A to Z and numbers from 0 to 9. It contains 2515 samples with variations signed by five different signers. The first and second signers signed 25 times each except alphabet T which has 20 samples, while the third and fourth signers signed five times each and the last signer signed ten times. Figure 7 displays the samples for each class.

**Figure 8:**
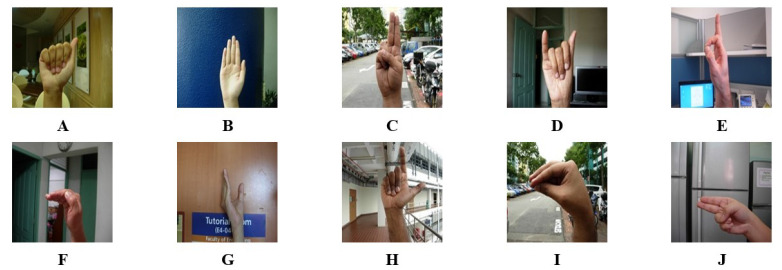
Image samples for each class from the NUS hand gesture dataset.

The NUS hand gesture dataset, proposed in [37], contains 10 hand gesture classes including alphabets from A to J, with a total of 2000 image samples. The dataset features 40 signers, with each signer signing five times for each class, resulting in a total of 200 samples per class to increase the variation in hand gestures. Figure 8 shows the image samples for each class in the dataset.

### 4.2. Experimental Setup

This section presents an experimental evaluation of the proposed solution using the same three datasets selected for benchmarking: the ASL dataset, ASL with digits dataset, and NUS hand gesture dataset. The transfer learning approach of pretraining Vision Transformer, as discussed earlier, will be implemented and evaluated.

Two validation techniques will be used in the experiments: holdout and k-fold cross-validation, specifically Leave-One-Out Cross-Validation (LOOCV). Holdout validation involves using 80% of the dataset for training and 20% for testing. LOOCV divides the dataset into *k* subsets, with each subset taking turns as the test set. This method reduces bias estimation and the chance of overfitting. In this study, we use 5-fold cross-validation, where the dataset is divided into five subsets, each taking turns as the training and testing set, as shown in Table 2.

### 4.3. Experimental Analysis

Experiments are conducted to determine the optimal configuration of the Vision Transformer for achieving the best performance across all three benchmark datasets. To prepare the data, all images are resized to 256×256. This is followed by a flatten and batch normalization layer. Then, a dense layer with a softmax activation function is added to output the predictions. Rectified Adam optimizer with a learning rate of 0.0001, categorical cross-entropy as the loss function, and accuracy as the evaluation metric, is utilised. In addition, early stopping with patience of 5 is employed. Furthermore, adaptive learning rate with factor of 0.2, patience of 2, minimum delta of 0.0001, and minimum learning rate of 0.000001 is adopted.

We compare the performance of the pre-trained base ViT with a patch size of 32×32 (ViT B32) using two different weights: ImageNet21k and ImageNet21k + ImageNet2012. To achieve maximum performance, all 19 layers were fine-tuned. The results show that the ViT pre-trained on ImageNet21k + ImageNet2012 outperforms the ViT pre-trained on ImageNet21k, across all three benchmark datasets. The ViT with ImageNet21k + ImageNet2012 weights achieves an impressive average testing accuracy of 99.97% for the ASL dataset, 99.01% for the ASL with digits dataset, 99.85% for the NUS hand gesture dataset, and an overall average accuracy of 99.61%. Therefore, the ViT with ImageNet21k + ImageNet2012 weights is selected for the following experiment, which involve fine-tuning the model.

The fine-tuning involves unfreezing layers of the pre-trained ViT model to train the model further. The results show that as more layers are unfrozen, the model’s performance improved. The proposed HGR-ViT achieves the highest performance when 12 layers are unfreezed, with an average testing accuracy of 99.98%, 99.36%, and 99.85% for ASL dataset, ASL with digits dataset, and NUS hand gesture dataset, respectively. An average of 99.73% accuracy is obtained across all three datasets.

### 4.4. Experimental Results and Discussions

This work aims to propose a Vision Transformer (ViT) model for static hand gesture recognition. ViT leverages the self-attention mechanism to capture complex relationships between image patches, which can better handle the similarity problem between hand gestures and variations in pose, lighting, background, and occlusions. Moreover, pre-trained ViT can reduce the data requirements during training, showing some ability to perform well with smaller datasets. Additionally, ViT can improve the interpretability of the hand gesture recognition system, generating attention maps that highlight the most important parts of an image for classification. These maps enable users to better understand why certain gestures are being recognized or not, improving the system’s transparency and accountability.

**Figure 9:**
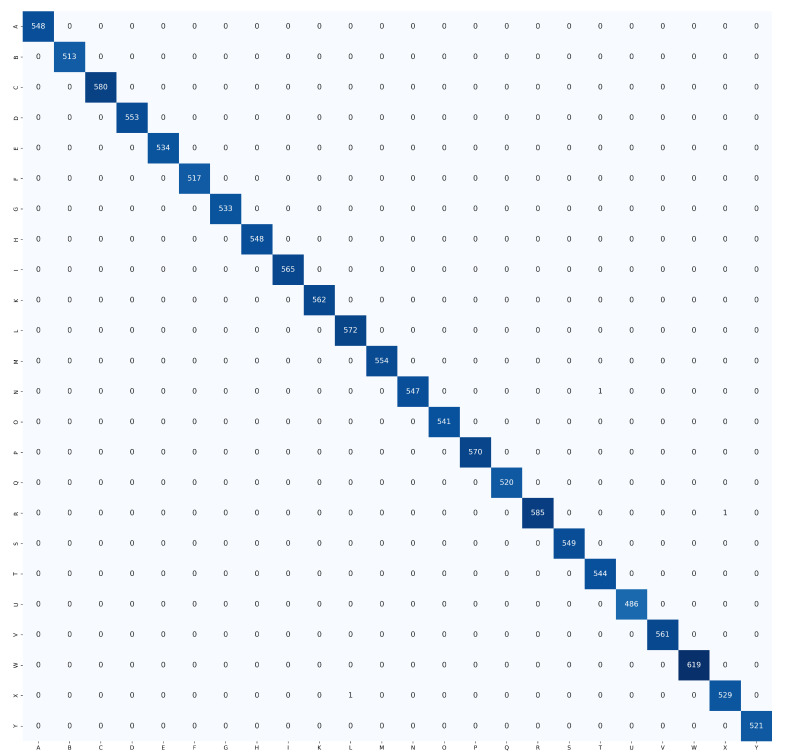
Confusion matrix for ASL dataset.

**Figure 10:**
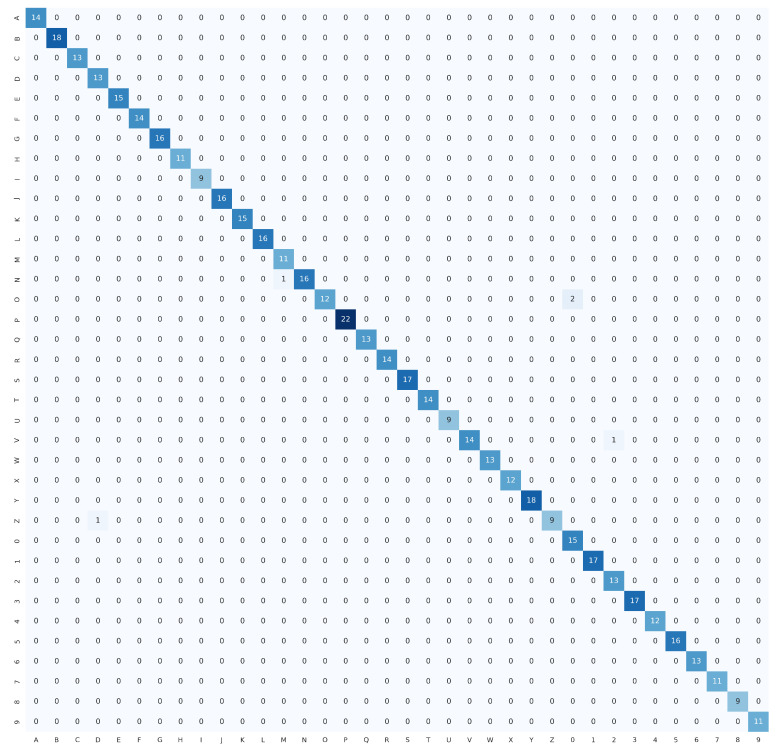
Confusion matrix for ASL with digits dataset.

**Figure 11:**
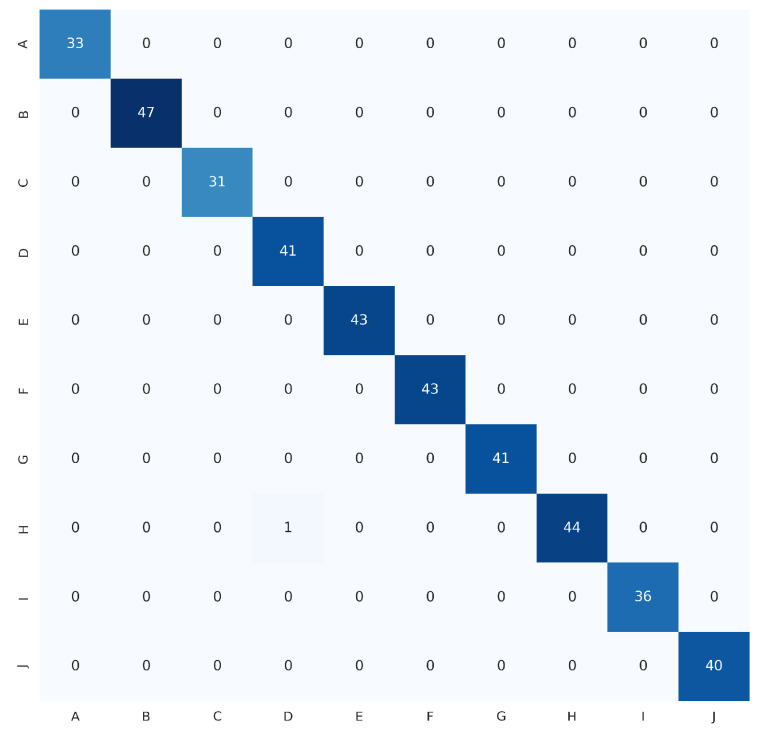
Confusion matrix for NUS hand gesture dataset.

The proposed HGR-ViT, which employs fine-tuning of a pre-trained Vision Transformer with ImageNet21k + ImageNet2012 weights, has demonstrated outstanding performance in static hand gesture recognition. The fine-tuning process was optimised with the unfreezing of 12 layers, using 256×256 image dimensions. Early stopping and adaptive learning rate were utilised to reduce overfitting and model error. Table 3 highlights the best results achieved by the proposed solution, demonstrating its superior performance over existing methods. In addition, confusion matrix of the ASL dataset, ASL with digits dataset, and NUS hand gesture dataset, are presented in Figure 9, Figure 10 and Figure 11, respectively.

**Figure 12:**
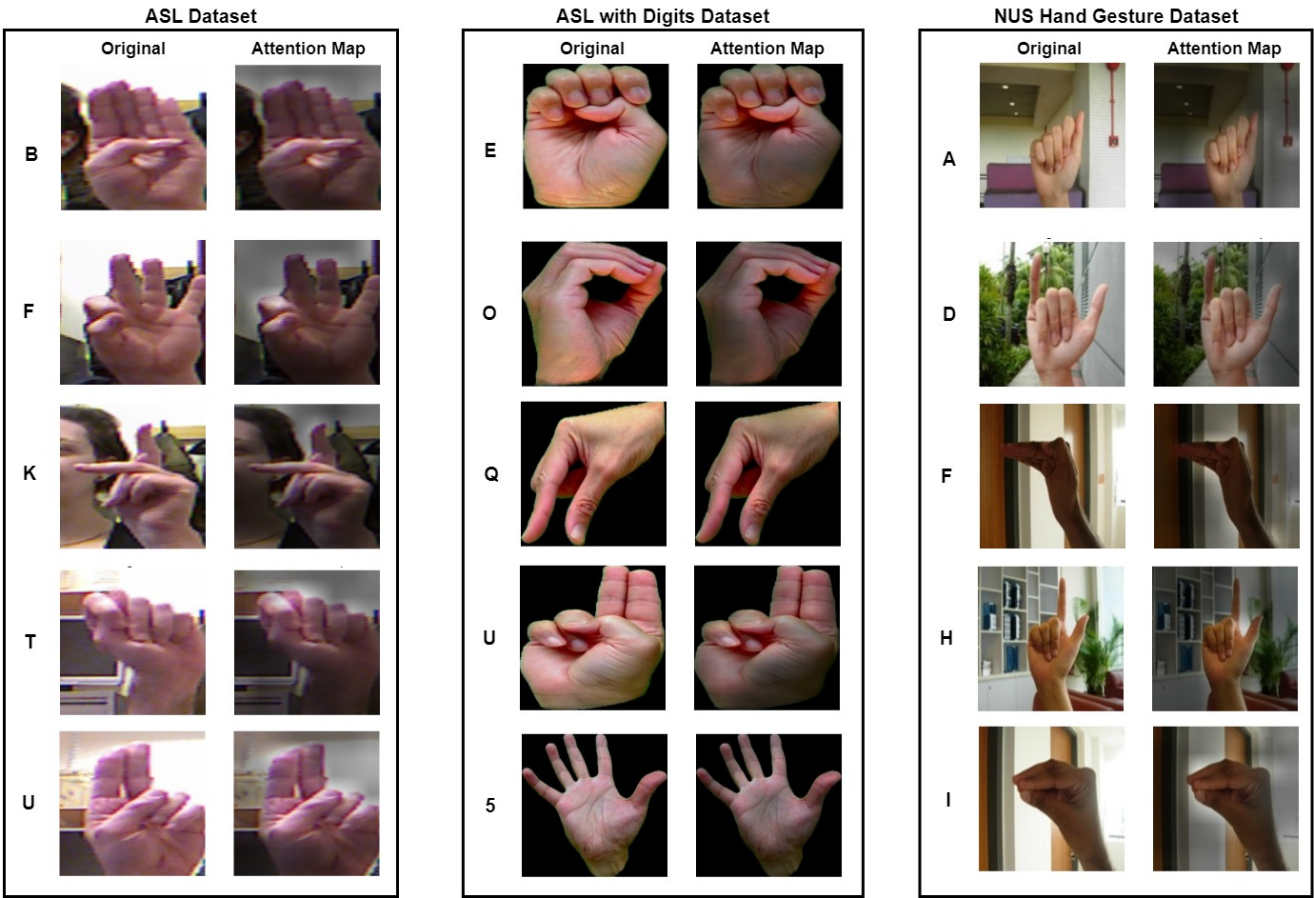
Sample attention map images for different alphabets in the benchmark datasets.

In addition to the quantitative performance evaluation, we also conducted a qualitative inspection of the proposed HGR-ViT. Figure 12 illustrates attention maps generated by the model for selected image samples from the three datasets. These attention maps demonstrate that the model is capable of focusing its attention on the relevant hand gestures while ignoring the background of the images. For instance, for the ASL dataset, the attention maps show that the model correctly identifies the hand gestures. In the case of the ASL with digits dataset, the model attends to the specific fingers that make up the hand gestures, thanks to the solid black background. Similarly, for the NUS hand gesture dataset, the attention maps reveal that the model focuses on the hand gestures and disregards the complex background.Overall, these results indicate that the proposed HGR-ViT performs well both quantitatively and qualitatively.

**Figure 13:**
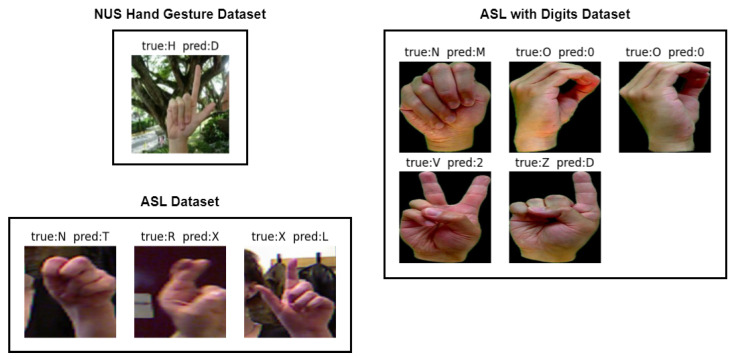
Sample of misclassified images for different alphabets in the benchmark datasets.

Figure 13 depicts misclassified images from the ASL dataset, ASL with digits dataset, and NUS hand gesture dataset, which were generated from the confusion matrix of Figure 9, Figure 10 and Figure 11, respectively. Three samples were misclassified in the ASL dataset: a hand gesture of class N was misclassified as class T, a hand gesture of class R was misclassified as class X, and a hand gesture of class X was misclassified as class L. The misclassification of hand gesture N as T can be attributed to the fact that these two classes share a similar appearance, with only slight variations in the thumb’s positioning, which can be difficult to distinguish even for humans in some angles. The misclassification of hand gesture R as X may be due to the slight blur in the image, which can lead to confusion between classes R and X, as they share some similarities. Finally, the misclassification of hand gesture X as L may be attributed to the fact that hand gestures X and L are similar in appearance, or it could be due to errors in image labeling by the authors.

As for ASL with digits dataset, there were five misclassified samples. One of them was the hand gesture of class N, which was misclassified as class M. This misclassification can be attributed to the similarity between the hand gestures, with only slight variations in the positioning of the thumb. Similarly, the hand gesture of class V that was misclassified as class 2 can also be attributed to the similarity in the hand gesture with slight variations in the positioning of the thumb. Another misclassification occurred for the hand gesture of class O, which was misclassified as class 0. This is due to the similarity between the two hand gestures, which might also be misclassified by humans if presented without context. Finally, the hand gesture of class Z was misclassified as class D, which might be due to the similarity between the hand gestures or errors in image labeling by the dataset authors. Overall, these misclassifications might occur due to the similarity between the hand gestures or errors in the dataset.

In the NUS hand gesture dataset, one misclassified sample was found where the hand gesture of class H was misclassified as class D. This misclassification might have been caused by the complex background of the image, where the model mistakenly identified the tree branches as fingers of the signer.

Table 4 presents the classification accuracy of various methods that were evaluated on the three benchmark datasets. The HGR-ViT method proposed in this research study demonstrates superior performance compared to all other existing methods across all three datasets, achieving the highest classification accuracy. This remarkable performance is attributed to the self-attention mechanism of the ViT model, which enables the model to capture complex relationships between image patches. The HGR-ViT approach surpasses conventional CNN models in this aspect and effectively addresses similarity issues between hand gestures, as well as variations in pose, lighting, background, and occlusions. This results in a significant improvement in accuracy, allowing the HGR-ViT method to outperform existing methods. The proposed method achieves a classification accuracy of 99.98% for the ASL dataset, 99.36% for the ASL with digits dataset, and 99.85% for the NUS hand gesture dataset. Moreover, the average classification accuracy across the three datasets is 99.73%. These results demonstrate the effectiveness of the proposed HGR-ViT method in hand gesture recognition and its superiority over other existing methods.

## 5. Conclusions

In conclusion, this paper proposes a fine-tuning of pre-trained Vision Transformer called HGR-ViT for static hand gesture recognition. The proposed method outperforms existing state-of-the-art CNN models, as demonstrated in the comparison with other methods. To prevent overfitting during the training process, early stopping and adaptive learning rate are utilised, contributing to the enhancement of the model’s performance. The experimental results show that the proposed method achieves better performance than existing approaches on three benchmark datasets. However, ViT requires a large amount of data to be trained on, which may not always be available in hand gesture recognition datasets. Additionally, ViT may not be able to capture fine-grained details in images, which could be important in recognising subtle differences between hand gestures. In terms of future research, one possible direction is to optimise the training process of ViT and reduce the amount of data required. The combination of ViT with other models such as CNNs can also improve recognition of fine-grained details. Lastly, researchers can also investigate ways to incorporate temporal information into hand gesture recognition systems, which can improve accuracy and robustness. Overall, the proposed approach provides a robust and effective solution for vision-based static hand gesture recognition.

## Figures and Tables

**Table 1:** Table of Summary of Related Works.

Category	Method	Reference
Hand-Crafted	SIFT feature extraction with Sparse Autoencoder neural network classification	[14]
	SIFT feature extraction with feedforward neural network classification	[22]
	HOG and SIFT feature extraction with K-Nearest Neighbour classification	[23]
	LBP and HOG feature extraction with AdaBoost classification	[24]
	HOG feature extraction with SVM and ANN classification	[15]
	DWT with F-ratio coefficient selection feature extraction with SVM classification	[25]
	DWT feature extraction with HMM classification	[16]
	Two-dimensional DWT feature extraction, SURF key point extraction and BOF feature space conversion with SVM classification	[26]
Deep Learning	RGB and Depth parallel CNN	[17]
	Smoothing and contrast enhancement, segmentation and delimitation and extraction of object of interest before classification using CNN	[27]
	Smoothing and disturbance removal, feature extraction with dilation and erosion before classification using CNN	[31]
	CNN with network initialisation and L2 regularisation	[28]
	Application of CNN with DAG network structure	[29]
	RGB and Depth parallel transfer learning VGG19	[1]
	Pre-trained AlexNet with optimisation using ABC algorithm	[2]
	Two-stage HGR: 1 CNN model trained for hand gesture recognition in first stage; 2 CNN model, 1 trained on segmented image, 1 trained on original image in second stage	[30]
	YOLOv3 with DarkNet-53 CNN	[4]
	CNN with spatial pyramid pooling (CNN-SPP)	[3]
	DenseNet with modified transition layer (EDenseNet)	[6]
	Network based on PointNet architecture	[32]

**Table 2:** Dataset distribution for training and testing sets.

Dataset	Folds	Training Set (80%)	Testing Set (20%)	Total (100%)
ASL	5	52,619	13,155	65,774
ASL with Digits	5	2012	503	2515
NUS Hand Gesture	5	1600	400	2000

**Table 3:** Performance of the proposed HGR-VIT on the three datasets.

Dataset	Cross Validation Set	Train Accuracy (%)	Test Accuracy (%)
ASL	1	100.00	99.98
	2	100.00	99.98
	3	100.00	99.98
	4	100.00	99.98
	5	100.00	99.99
	Average	100.00	99.98
ASL with digits	1	100.00	99.20
	2	100.00	99.60
	3	100.00	99.80
	4	100.00	99.20
	5	100.00	99.01
	Average	100.00	99.36
NUS hand gesture	1	100.00	99.50
	2	100.00	99.75
	3	100.00	100.00
	4	100.00	100.00
	5	100.00	100.00
	Average	100.00	99.85

**Table 4:** Classification accuracy (%) of the state-of-the-art methods and the proposed HGR-ViT on the three hand gesture datasets.

Method	ASL Dataset	ASL with Digits Dataset	NUS Hand Gesture Dataset	Average Accuracy
CNN Baseline A [27]	99.85	98.69	89.15	95.90
CNN Baseline B [31]	99.78	98.65	89.30	95.91
ADCNN [28]	98.50	98.49	83.10	93.36
DAG-CNN [29]	99.89	98.13	91.05	96.36
EdenseNet [6]	99.89	98.85	96.75	98.50
CNN-SPP [3]	99.94	99.17	95.95	98.35
HGR-ViT (Ours)	99.98	99.36	99.85	99.73

## Data Availability

Not applicable.
